# A smart way back to work – Einfluss digitaler Rehabilitation auf die Arbeitsfähigkeit nach Handverletzungen

**DOI:** 10.1007/s00132-026-04814-z

**Published:** 2026-03-31

**Authors:** Simon Bauknecht, Daniel Vergote, Martin Mentzel, Diego Chiquiza, Michael Lebelt, Richard-Tobias Moeller

**Affiliations:** 1 Klinik f. Unfall‑, Hand‑, Plastische u. Wiederherstellungschirurgie, Universitätsklinikum Ulm, Albert-Einstein-Allee 23, 89081 Ulm, Deutschland; 2 Klinik f. Hand- u. Plastische u. Ästhetische Chirurgie, Klinikum Stuttgart, Stuttgart, Deutschland

**Keywords:** Künstliche Intelligenz, Knochenfrakturen, Handverletzungen, Therapietreue, Mobile Anwendungen, Artificial intelligence, Bone fractures, Hand injuries, Patient compliance, Mobile applications

## Abstract

**Hintergrund:**

Die Wiederherstellung der Arbeitsfähigkeit nach Handverletzungen ist ein zentrales Ziel der Rehabilitation. Digitale Therapieanwendungen halten zunehmend Einzug in den klinischen Alltag. Bisher fehlen jedoch Daten zum Einfluss solcher Technologien auf alltagsrelevante Parameter, wie die Dauer der Arbeitsunfähigkeit. Insbesondere ist unklar, ob eine App-gestützte Handtherapie die Rückkehr in den Beruf beschleunigen kann.

**Fragestellung:**

Ziel der Studie war es, den Effekt einer Handtherapie-App zusätzlich zur Standardversorgung auf die Dauer der Arbeitsunfähigkeit im Vergleich zur alleinigen Standardversorgung zu untersuchen.

**Material und Methoden:**

In einer prospektiven, kontrollierten Studie wurden 105 Patienten mit operativ versorgten Handverletzungen eingeschlossen. Die Kontrollgruppe erhielt eine standardisierte Nachbehandlung mit Handtherapie. Die Interventionsgruppe absolvierte zusätzlich über 90 Tage ein App-basiertes Trainingsprogramm mit individualisiertem, KI-gestütztem Übungsplan, Gamification-Elementen und visueller Verlaufskontrolle. Als primärer Endpunkt wurde die Anzahl der Krankheitstage bis zur beruflichen Wiedereingliederung erfasst.

**Ergebnisse:**

Patienten der Interventionsgruppe wiesen signifikant weniger Krankheitstage auf als die Patienten der Kontrollgruppe. Hinsichtlich der Indikation erfolgte bei Patienten mit Mittelhandfrakturen die Rückkehr in das Berufsleben in der Interventionsgruppe signifikant früher als in der Kontrollgruppe. Zusätzlich zeigte sich ein signifikanter Effekt der Handtherapie-App auf die Dauer der Arbeitsunfähigkeit bei Patienten mit Beeinträchtigung der nichtdominanten Hand und bei Patienten, die keine handwerklichen Berufe ausüben.

**Schlussfolgerung:**

Die Ergebnisse deuten darauf hin, dass eine App-gestützte Handrehabilitation die Dauer der Arbeitsunfähigkeit verkürzen kann. Besonders bei Mittelhandfrakturen bietet der digitale Therapieansatz eine vielversprechende Ergänzung zur Standardversorgung. Weitere Studien mit größeren Fallzahlen sind erforderlich, um die Effekte für andere Indikationen zu verifizieren.

## Abstract (en)

**Background:**

Restoring the ability to work after hand injuries is a central goal of rehabilitation. Digital therapy applications are increasingly finding their way into everyday clinical practice. However, data on the influence of such technologies on parameters relevant to everyday life, such as the duration of incapacity to work, is still lacking. In particular, it is unclear whether app-supported hand therapy can accelerate the return to work.

**Objective:**

The aim of the study was to investigate the effect of a hand therapy app in addition to standard care on the duration of incapacity to work compared to standard care alone.

**Material and methods:**

In a prospective, controlled study, 105 patients with surgically treated hand injuries were included. The control group received standardized follow-up treatment with hand therapy; the intervention group additionally completed an app-based training program over 90 days with an individualized, AI-supported exercise plan, gamification elements and visual progress monitoring. The primary endpoint was the number of sick days until return to work.

**Results:**

Patients in the intervention group had significantly fewer sick days than patients in the control group. With regard to the indication, patients with metacarpal fractures returned to work significantly earlier in the intervention group than in the control group. In addition, there was a significant effect of the hand therapy app on the duration of incapacity to work in patients with impairment of the non-dominant hand and in patients who do not carry out manual occupations.

**Conclusion:**

The results indicate that app-supported hand rehabilitation can shorten the duration of incapacity for work. For metacarpal fractures in particular, the digital therapy approach offers a promising addition to standard care. Further studies with larger case numbers are required to verify the effects for other indications.

## Hinführung zum Thema

Handverletzungen gehen häufig mit erheblichen funktionellen Einschränkungen einher. Neben der funktionellen Wiederherstellung spielt eine möglichst schnelle Rückkehr in das Berufsleben eine zentrale Rolle. Während digitale Rehabilitationsansätze zunehmend an Bedeutung gewinnen, ist ihr Nutzen im klinischen Alltag bislang nur unzureichend belegt. In dieser Studie wurde der Effekt einer Handtherapie-App auf die berufliche Wiedereingliederung von Patienten mit Handverletzungen erstmalig quantifiziert – ein Aspekt mit hoher praktischer und gesundheitsökonomischer Relevanz.

## Hintergrund

Handverletzungen zählen zu den häufigsten Diagnosen in der unfallchirurgischen Notfallversorgung [1]. Aufgrund ihrer hohen Inzidenz und der zentralen Rolle der Hand in nahezu allen beruflichen Tätigkeiten [2] stellen sie eine wesentliche Ursache sowohl für vorübergehende als auch für langanhaltende Einschränkungen der Arbeitsfähigkeit dar [3]. Die rasche Rückkehr ins Erwerbsleben ist ein zentrales Ziel in der muskuloskelettalen Rehabilitation – sowohl aus individueller als auch aus gesamtgesellschaftlicher Perspektive. Eine verzögerte Wiedereingliederung geht häufig mit erheblichen sozioökonomischen Belastungen einher. Wachter et al. (2005) berichten beispielsweise von durchschnittlich 78 Tagen (ca. 11 Wochen) Arbeitsunfähigkeit, hauptsächlich bei Finger- und Mittelhandverletzungen [3]. Laut Bundesanstalt für Arbeitsschutz und Arbeitsmedizin (BAuA) belaufen sich die Kosten pro Tag Arbeitsunfähigkeit je nach Branche auf 74–251 € [4].

Besonders bei Patienten mit Handverletzungen können schon geringe Funktionseinschränkungen die berufliche Leistungsfähigkeit erheblich beeinträchtigen [5, 6]. Häufig sind es junge berufstätige Erwachsene, die durch Arbeits- oder Freizeitunfälle betroffen sind [7]. Die operative Therapie richtet sich nach Lokalisation und Schweregrad der Verletzung [7–9] und erfordert häufig eine postoperative temporäre Immobilisation von mehreren Tagen bis Wochen. Diese notwendige Ruhigstellung begünstigt jedoch die Bildung von Adhäsionen und kann zusätzliche funktionelle Defizite verursachen [10–12]. Für eine erfolgreiche Rehabilitation ist eine frühzeitige, strukturierte und intensive handtherapeutische Nachbehandlung unerlässlich [3, 13]. Neben der standardmäßigen Versorgung durch Physio- und Ergotherapie spielt das eigenverantwortliche Üben zu Hause eine entscheidende Rolle [14, 15]. Obwohl die Wirksamkeit solcher Heimtrainingsprogramme gut dokumentiert ist, bleibt die tatsächliche Umsetzung im Alltag – insbesondere hinsichtlich der Therapietreue – schwer messbar. Digitale Therapieanwendungen bieten hier neue Möglichkeiten: Sie können Übungen nachvollziehbarer machen, individuelle Fortschritte objektiv darstellen und durch spielerische Elemente die Motivation zur regelmäßigen Durchführung steigern [16–19].

Erste Studien zeigen positive Effekte auf den Bewegungsumfang und die Handfunktion nach Handverletzungen [17, 20–22]. Inwieweit der ergänzende Einsatz App-basierter Therapien auch gesundheitsökonomisch relevante Parameter wie die Dauer der Arbeitsunfähigkeit beeinflussen kann, war bislang noch unklar.

Ziel dieser Studie war es daher zu untersuchen, ob der Einsatz einer individualisierten, KI-gestützten Handtherapie-App zusätzlich zur Standardversorgung im Vergleich zur alleinigen Standardversorgung die Dauer der Arbeitsunfähigkeit nach operativ versorgten Handverletzungen verkürzen kann.

## Material und Methoden

### Studienpopulation

Eingeschlossen wurden Patienten im Alter von 18–65 Jahren mit operativ behandelten Handverletzungen, darunter Mittelhand- (ICD S62.2, S62.3) und Fingerfrakturen (ICD S62.4, S62.5, S62.6, S62.7), Beuge- (ICD S66.1, S66.6) sowie Strecksehnenverletzungen (ICD S66.3, S66.7). Voraussetzung war der Besitz eines Smartphones, ausreichende Deutschkenntnisse und die Bereitschaft zur Nutzung der Handtherapie-App Novio Hand (LIME medical GmbH, Mainz, Deutschland). Ausschlusskriterien waren neurologische Erkrankungen, akute Infektionen, ausgeprägte Hautveränderungen, Arthrosen, komplexe Verletzungsmuster mit Nerven- oder Gefäßschäden sowie fehlende Teilnahme an den Erhebungszeitpunkten.

Die Rekrutierung erfolgte über ein Jahr (ab Dezember 2021). Insgesamt wurden 119 Patienten eingeschlossen und in zwei Gruppen randomisiert: Kontrollgruppe (KG; *n* = 51) und Interventionsgruppe (IG; *n* = 54). Vierzehn Patienten waren nicht berufstätig; somit konnten 105 Datensätze ausgewertet werden (KG: *n* = 51; Durchschnittsalter 35,2 Jahre ± 14; 37 männlich/14 weiblich; IG: *n* = 54; Durchschnittsalter 40,7 Jahre ± 16,9; 37 männlich/17 weiblich).

### Interventionsmaßnahmen

Die in der Studie verwendete KI-basierte Handtherapie-App Novio Hand ist ein CE-zugelassenes Medizinprodukt (MDR Klasse I), das speziell für die häusliche Rehabilitation entwickelt wurde. Die multimodale App basiert auf drei Säulen: Bewegungstherapie, Narbenmassage sowie Gamification- und Motivationsmodule. Bewegungsübungen wurden zusammen mit zertifizierten Experten für Handtherapie der DAHTH (Handtherapie Praxis Mack, Ulm), entwickelt und sind indikationsspezifisch auf Frakturen sowie Sehnenverletzungen abgestimmt. Die App nutzt die Smartphone-Frontkamera zur Echtzeiterfassung der Bewegungsabläufe. Eine KI analysiert das Bewegungsausmaß (ROM) und die Qualität der Ausführung, passt Übungen individuell an den Behandlungsfortschritt an und gibt in Echtzeit unmittelbares Feedback. Vor und nach jeder Übung wird die Schmerzintensität via Visuelle Analog Skala (VAS) erfasst; ein weiterer Algorithmus modifiziert daraufhin automatisch Trainingsumfang und -intensität. Zusätzlich enthält die App edukative Videos zum Verletzungsbild und Rehabilitationsziel sowie ein Gamification-Modul: Bestimmte Übungen sind in interaktive Spielsequenzen eingebettet (z. B. Steuerung einer Spielfigur). Push-Benachrichtigungen, Belohnungssysteme und Fortschrittsvisualisierungen sollen die Motivation weiter steigern.

### Studienablauf

Es handelt sich um eine prospektive, randomisierte kontrollierte Studie im Prä‑/Post-Design. Nach Aufklärung erhielten alle Patienten eine standardisierte handchirurgische Versorgung sowie eine indikationsspezifische Ruhigstellung. Nach Freigabe begann eine 12-wöchige aktive Rehabilitationsphase. Die Kontrollgruppe erhielt zusätzlich zur Standardversorgung (SoC; 18 Einheiten Handtherapie à 2- bis 3‑mal pro Woche) keine weitere Intervention. Die Interventionsgruppe nutzte zusätzlich zur SoC die App Novio Hand. Die Dauer der Arbeitsunfähigkeit wurde anhand der Dauer der ärztlichen bescheinigten Arbeitsunfähigkeit dokumentiert.

### Datenauswertung

Statistische Analysen erfolgten mit R (Version 4.4.3) für die Gesamtstichprobe und indikationsspezifisch sowie hinsichtlich der Einflussfaktoren (1) betroffene Hand (dominante Hand betroffen/nichtdominante Hand betroffen) oder (2) berufliche Tätigkeit (körperliche schwere Tätigkeit/belastungsarme Tätigkeit). Die Verteilung der Daten wurde mittels Shapiro-Wilk-Test geprüft; bei Normalverteilung kam ein t‑Test oder eine ANOVA zum Einsatz, ansonsten der Wilcoxon-Rangsummentest. Das Signifikanzniveau lag bei *p* ≤ 0,05. Effektstärken wurden entsprechend berechnet: Cohen’s d für t‑Tests, partielle Eta^2^ für ANOVA sowie Rangkorrelationskoeffizient r für den Wilcoxon-Test.

## Ergebnisse

Insgesamt wurden 105 Patienten in die Analyse eingeschlossen (KG: 51, IG: 54). Die Verteilung der Patienten auf die vier untersuchten Indikationen (Mittelhandfraktur, Fingerfraktur, Beugesehnenverletzung, Strecksehnenverletzung) ist in Tab. 1 dargestellt.

**Tab. 1:** Patientencharakteristiken

Charakteristiken		Interventionsgruppe	Kontrollgruppe	Gruppenunterschied
Gesamtpopulation	*n*	54	51	–
Geschlecht	Weiblich	17	14	*p* = 0,81
	Männlich	37	37	
Alter (Jahre)	MW ± SD	40,7 ± 16,9	35,2 ± 14,0	*p* = 0,08
*Indikationen*				
Mittelhandfrakturen	S62.3, S62.4	14	14	*p* = 0,99
Fingerfrakturen	S62.2, S62.6, S62.7	16	15	
Beugesehnenverletzungen	S66.1, S66.6	11	11	
Strecksehnenverletzungen	S66.3, S66.7	13	11	

Tab. 2 zeigt die deskriptiven Statistiken zur Anzahl der Krankheitstage (Arbeitsunfähigkeit) für beide Gruppen – sowohl indikationsübergreifend als auch indikationsspezifisch.

**Tab. 2:** Anzahl der Krankheitstage

Anzahl der Krankheitstage	*n*	MW ± SD	Median	Min	Max
*Kontrollgruppe gesamt*	51	56,5 ± 32,5	52	7	162
Mittelhandfraktur	14	48,4 ± 23,1	50	14	93
Fingerfraktur	15	41,7 ± 19,2	42	7	78
Beugesehnenverletzung	11	80,5 ± 39,8	79	13	142
Strecksehnenverletzung	11	63,2 ± 37,3	56	35	162
*Interventionsgruppe gesamt*	54	43,8 ± 28,7	38	0	115
Mittelhandfraktur	14	27,2 ± 17,2	24,5	7	63
Fingerfraktur	16	30,9 ± 21,5	30	0	73
Beugesehnenverletzung	11	73,8 ± 26,3	75	35	115
Strecksehnenverletzung	13	52,1 ± 26,7	53	23	99

### Gesamtanalyse: App-Effekt auf die Dauer der Arbeitsunfähigkeit

**Abb. 1:**
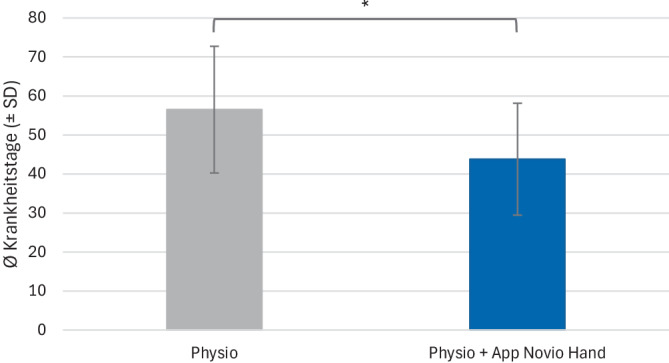
Analyse der Arbeitsunfähigkeit der Gesamtpopulation: Anzahl der Krankheitstage (MW ± SE) für die Kontrollgruppe Physio (*grau*) und die Interventionsgruppe Physio + App Novio Hand (*blau*). *MW* Mittelwert, *SE* Standardfehler, *Stern* zeigt *p* < 0,05

Für den Vergleich Anzahl der Krankheitstage zwischen Patienten der IG und Patienten der KG wurde aufgrund der nicht normalverteilten Daten der Wilcoxon-Test durchgeführt. Analysen der Krankheitstage zeigten eine signifikant (W = 1704, *p* = 0,03, r = 0,204) um durchschnittlich 13 Tage kürzere Dauer der Arbeitsunfähigkeit der IG (43,8 ± 28,7) im Vergleich zur KG (56,5 ± 32,5). Dies verdeutlicht den positiven Einfluss der Handtherapie-App Novio Hand auf den Rehabilitationsverlauf (Abb. 1). Die Dauer der Arbeitsunfähigkeit konnte durch die Heimübungen mittels App um durchschnittlich 22 % reduziert werden.

#### Einfluss der beeinträchtigten Hand

**Abb. 2:**
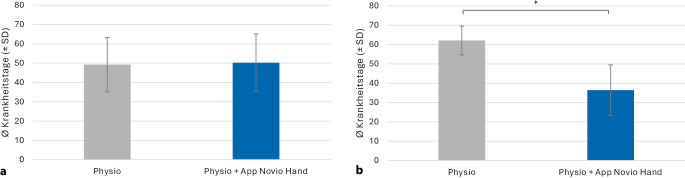
Analyse der Arbeitsunfähigkeit der Gesamtpopulation hinsichtlich der betroffenen Hand (dominante [**a**]/nichtdominante Hand [**b**]): Anzahl der Krankheitstage (MW ± SE) für die Kontrollgruppe Physio (*grau*) und die Interventionsgruppe Physio + App Novio Hand (*blau*). *MW* Mittelwert, *SE* Standardfehler, *Stern* zeigt *p* < 0,05

Eine zweifaktorielle Varianzanalyse (ANOVA) wurde durchgeführt, um den Einfluss von Gruppenzugehörigkeit (KG vs. IG) und betroffener Hand (dominant vs. nichtdominant) auf die Dauer der Arbeitsunfähigkeit zu untersuchen. Die Analyse zeigte einen signifikanten Haupteffekt der Gruppe: Patienten in der IG hatten im Durchschnitt weniger Krankheitstage, F(1, 99) = 5,15; *p* = 0,026; η^2^ₚ = 0,047. Der Haupteffekt der betroffenen Hand war nicht signifikant (F[1, 99] = 0,01; *p* = 0,907; η^2^ₚ = 0,000). Allerdings gab es eine signifikante Wechselwirkung zwischen Gruppe und betroffener Hand: F(1, 99) = 5,02; *p* = 0,027; η^2^ₚ = 0,046. Das bedeutet, dass der Effekt der App-Intervention von der Hand abhängt (Abb. 2). Post-hoc-Vergleiche mit Bonferroni-Korrektur ergaben: Bei Patienten mit betroffener nichtdominanter Hand war die IG deutlich kürzer krank (36,41 ± 26,14 Tage) als die KG (62,10 ± 34,93 Tage; *p* = 0,001). Bei Patienten mit betroffener dominanter Hand gab es keinen Unterschied zwischen den Gruppen (*p* = 0,905).

#### Einfluss der beruflichen Tätigkeit

Auch beim Einfluss der beruflichen Tätigkeit wurde eine zweifaktorielle ANOVA durchgeführt, um den Einfluss von Gruppe (KG vs. IG) und Beruf (schwere vs. körperlich nicht belastende Tätigkeit) auf die Dauer der Krankheit zu untersuchen.

Die Ergebnisse zeigten:

- Ein signifikanter Effekt der Gruppe: Patienten in der IG waren im Durchschnitt kürzer krank (F[1,96] = 6,54; *p* = 0,012; η^2^ₚ = 0,064).
- Ein signifikanter Effekt des Berufs: Patienten mit einer belastungsarmen Tätigkeit waren signifikant kürzer krank als Patienten mit körperlich schwerer Tätigkeit (F[1,96] = 19,80; *p* < 0,001; η^2^ₚ = 0,171).
- Es gab keine signifikante Wechselwirkung zwischen Gruppe und Beruf (F[1,96] = 1,10; *p* = 0,297).

**Abb. 3:**
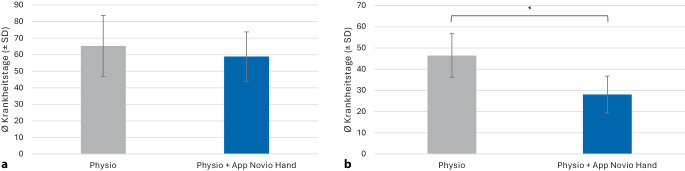
Analyse der Arbeitsunfähigkeit der Gesamtpopulation hinsichtlich der beruflichen Tätigkeit (körperlich schwere Tätigkeit [**a**]/belastungsarme Tätigkeit [**b**]): Anzahl der Krankheitstage (MW ± SE) für die Kontrollgruppe Physio (*grau*) und die Interventionsgruppe Physio + App Novio Hand (*blau*). *MW* Mittelwert, *SE* Standardfehler, *Stern* zeigt *p* < 0,05

Post-hoc-Analysen mit Bonferroni-Korrektur ergaben: Bei Patienten mit einer belastungsarmen Tätigkeit kehrten die IG-Patienten signifikant früher in den Beruf zurück als die KG-Patienten (IG: 28,07 ± 17,34 Tage; KG: 46,42 ± 20,63 Tage; *p* = 0,033, Abb. 3).

### Subgruppenanalyse: App-Effekt auf Krankheitstage

#### Mittelhandfrakturen

**Abb. 4:**
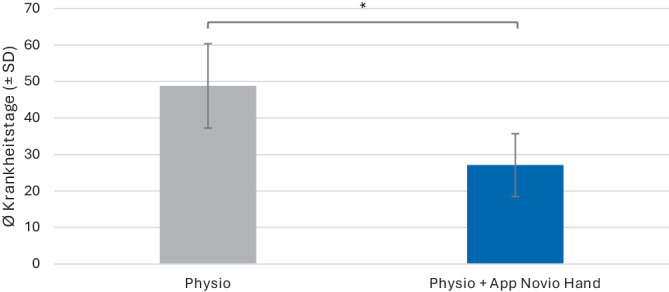
Analyse der Arbeitsunfähigkeit bei Patienten mit Mittelhandfrakturen: Anzahl der Krankheitstage (MW ± SE) für die Kontrollgruppe Physio (*grau*) und die Interventionsgruppe Physio + App Novio Hand (*blau*). *MW* Mittelwert, *SE* Standardfehler, *Stern* zeigt *p* < 0,05

Bei Patienten mit einer Mittelhandfraktur wies die IG mit 27,1 ± 17,2 Krankheitstagen eine um 44 % signifikant kürzere Arbeitsunfähigkeit auf als bei Patienten der KG (48,8 ± 23,1 Krankheitstage). Der Unterschied war statistisch signifikant (t = 2,75, df = 24,01, *p* = 0,01) (Abb. 4). Die Krankheitsdauer der IG war sowohl bei den Patienten mit betroffener dominanter Hand als auch betroffener nichtdominanter Hand kürzer als die Krankheitsdauer der KG. Diese Ergebnisse waren jedoch statistisch nicht signifikant. Auch die Krankheitsdauer bei Patienten der IG mit körperlich schwerer Tätigkeit sowie belastungsarmer Tätigkeit war kürzer als bei KG Patienten, aber ebenfalls ohne statistische Signifikanz.

#### Fingerfrakturen

Die durchschnittliche Arbeitsunfähigkeit bei Patienten mit einer Fingerfraktur betrug 30,9 ± 21,5 Tage in der IG gegenüber 41,7 ± 19,2 Tagen in der KG (t = 1,47, df = 28,93, *p* = 0,15) was einer Reduktion der Dauer der Arbeitsunfähigkeit um ca. ein Viertel entspricht. Auch in der Subgruppe mit betroffener nichtdominanter Hand wies die IG weniger Krankheitstage auf als die KG (nicht signifikant). Ein ähnliches Bild zeigt sich bei Patienten mit belastungsarmer Tätigkeit: Diese kehrten unter Einsatz der Handtherapie-App früher an den Arbeitsplatz zurück als die KG. Bei Patienten mit körperlich schwerer Tätigkeit zeigte sich kein entsprechender Effekt.

#### Beugesehnenverletzungen

Auch bei Patienten mit einer Beugesehnenverletzung konnte eine um ca. 1 Woche verkürzte Arbeitsunfähigkeit in der IG im Vergleich zur KG festgestellt werden, jedoch ohne statistische Signifikanz. Die mittlere Arbeitsunfähigkeit betrug 73,8 ± 26,3 Tage in der IG und 80,5 ± 39,8 Tage in der KG. Es zeigte sich kein Unterschied von IG und KG sowohl hinsichtlich der betroffenen Hand (betroffene dominante/nichtdominante Hand) als auch hinsichtlich der beruflichen Tätigkeit.

#### Strecksehnenverletzungen

Bei Patienten mit einer Strecksehnenverletzung lag die durchschnittliche Arbeitsunfähigkeit in der IG bei 52,1 ± 26,7 Tagen ebenfalls um ca. 18 % niedriger als in der KG bei 63,2 ± 37,3 Tagen (nicht signifikant). Es zeigte sich ebenfalls kein signifikanter Einfluss der betroffenen Hand (betroffene dominante/nichtdominante Hand) oder beruflichen Tätigkeit.

## Diskussion

### Gesamtanalyse

Die vorliegende Studie zeigt, dass der Einsatz einer individualisierten, App-basierten Handtherapie (Novio Hand) in Ergänzung zur konventionellen Behandlung den Rehabilitationsverlauf bei operativ versorgten Handverletzungen deutlich verbessern kann. Besonders profitieren Patienten, wenn die nichtdominante Hand betroffen ist oder sie in körperlich belastungsarmen Berufen arbeiten. Insgesamt unterstreichen die Ergebnisse den positiven Einfluss digital unterstützter Rehabilitationsverfahren auf die Verkürzung der Arbeitsunfähigkeit [23]. Der signifikant kürzere Zeitraum der Arbeitsunfähigkeit in der IG weist auf den klinischen und gesundheitsökonomischen Mehrwert dieser Therapieform hin [24]. Da Arbeitsunfähigkeit mit erheblichen Kosten verbunden ist – laut BAuA zwischen 74 und 251 € pro Tag [4] – könnten bereits moderate Reduktionen der Krankheitsdauer wirtschaftlich bedeutsam sein [24]. Die in unserer Studie festgestellte durchschnittliche Reduktion um ca. 13 Krankheitstage würde somit zu einer Kostenersparnis von mindestens 962–18.574 €, insbesondere bei der häufig jungen und berufstätigen Zielgruppe, führen. Es ist jedoch wichtig zu beachten, dass die Effektstärke bei bestimmten Subgruppen – etwa bei Patienten mit Mittelhandfrakturen – besonders ausgeprägt war. Da bei diesen Frakturen bereits sehr früh mit der App-gestützten Handtherapie begonnen werden konnte [15], zeigt sich hier auch das Potenzial einer gezielten, strukturierten Handrehabilitation mittels App auch am stärksten. Bei Fingerfrakturen zeigte sich nur ein Trend zugunsten der App-Nutzer, was möglicherweise an kleineren Fallzahlen und der späteren Intervention im Vergleich zu Mittelhandfrakturen liegen könnte [25]. Bei Verletzungen an Beuge- oder Strecksehnen konnte kein signifikanter Effekt nachgewiesen werden. Dies könnte daran liegen, dass es bislang nur bedingt möglich ist, dass Sehnenverletzungen frühzeitig durch den Patienten selbstständig aktiv beübt werden können [26]. Gleichzeitig wirken sich Adhäsionen bei Sehnenverletzungen besonders stark aus [10]. Die längere Immobilisationszeit und die Notwendigkeit eines restriktiveren Belastungsaufbaus könnten die Wirkung von Heimübungsprogrammen begrenzen. Der potenzielle Nutzen digitaler Therapie in dieser Patientengruppe könnte sich erst über längere Zeiträume hinweg zeigen und blieb im vorliegenden Studienzeitraum möglicherweise unentdeckt. Hier könnten längere Beobachtungszeiträume notwendig sein, um potenzielle Langzeiteffekte zu erfassen.

Ein wichtiger Aspekt ist die Rolle der Handdominanz: Dass die dominante Hand im Alltag ohnehin stärker trainiert wird, erklärt den begrenzten Erfolg der App bei diesen Patienten. Im Gegensatz dazu können Patienten mit betroffener nichtdominanter Hand von der gezielten Unterstützung profitieren, da diese im Alltag meist weniger gefordert wird und Einschränkungen besser kompensiert werden können. Zudem sind in körperlich belastungsarmen Berufen – etwa bei Bürotätigkeit – oft flexiblere Arbeitsmodelle möglich, was eine frühere Rückkehr an den Arbeitsplatz erleichtert [27]. Durch den Einsatz der App wird dieser Effekt vermeintlich verstärkt. Zukünftige Studien sollten untersuchen, inwiefern auch körperlich schwer arbeitende Patienten besser von digitalen Rehabilitationsansätzen profitieren können, diesbezüglich sind noch weitere Forschungsarbeiten notwendig. Größere Stichproben wären zudem wünschenswert. Abschließend lässt sich sagen: Eine KI-gestützte App wie Novio Hand zur strukturierten, individualisierten Handrehabilitation stellt eine vielversprechende Ergänzung zur konventionellen Therapie dar. Sie kombiniert edukative Inhalte, Bewegungsfeedback sowie motivierende Elemente wie Gamification-Komponenten, die nachweislich die Adhärenz und funktionelle Ergebnisse verbessern können [16, 17, 20].

### Ausblick

Die vorliegenden Ergebnisse unterstreichen das Potenzial digitaler, patientenzentrierter Rehabilitationsansätze, um die Behandlungsergebnisse bei Handverletzungen nachhaltig zu verbessern [16, 17]. Die Integration von individualisierten Apps wie Novio Hand in den klinischen Alltag kann nicht nur die Dauer der Arbeitsunfähigkeit verkürzen, sondern auch die Therapietreue und Motivation der Patienten steigern.

Zukünftige Forschungsarbeiten sollten sowohl die Langzeitwirkungen solcher digitalen Interventionen untersuchen als auch prüfen, ob eine modulare, individuell an die Verletzungsmuster anpassbare App auch bei Patientengruppen mit bislang geringerer Effektstärke bei der Reduktion der Arbeitsunfähigkeitsdauer signifikante Verbesserungen erzielen kann. Zudem ist es entscheidend, digitale Therapien gezielt weiterzuentwickeln, um deren Wirksamkeit auch in Abhängigkeit individueller Bedürfnisse und Verletzungsmuster zu optimieren.

Technologisch eröffnen sich spannende Perspektiven: durch die Einbindung von KI zur individualisierten Anpassung der Trainingsprogramme, lässt sich die Effektivität und Akzeptanz digitaler Rehabilitation weiter steigern. Für die klinische Praxis bedeutet dies, dass digitale Rehabilitationsansätze künftig als integraler Bestandteil eines multimodalen Behandlungskonzepts etabliert werden sollten. Sie bieten die Chance, Ressourcen effizienter zu nutzen, individuelle Therapiepläne flexibler zu gestalten und somit eine patientenzentrierte Versorgung auf höchstem Niveau sicherzustellen [17, 28, 29].

Zudem erscheint es sinnvoll, die App dahingehend zu erweitern, dass Physiotherapeuten und Ärzte bereits während der Therapie Einblicke in den Fortschritt des Patienten erhalten [17, 18]. Dadurch könnte der Therapieverlauf noch gezielt angepasst und gesteuert werden. Selbst für die berufsgenossenschaftlichen Träger der gesetzlichen Unfallversicherung bietet die App spannende Möglichkeiten: Sie ermöglicht auch ihnen einen aktuellen Überblick über die Wirksamkeit laufender Therapien und den Behandlungsfortschritt. Darüber hinaus könnte zukünftig eine direkte Kommunikation per Nachrichtenfunktion zwischen Ärzten, Physiotherapeuten und Berufsgenossenschaften etabliert werden, was eine völlig neue Art der direkten und unbürokratischen Interaktion darstellen würde. Die erleichterte Kostenübernahme digitaler Handrehabilitation erscheint vor dem Hintergrund aktueller Evidenz als konsequenter nächster Schritt hin zu einer modernen, patientenzentrierten Versorgung. Eine strukturierte Integration in die Regelversorgung könnte langfristig nicht nur die Ergebnisqualität verbessern, sondern auch gesundheitsökonomische Ressourcen sinnvoll nutzen. Unsere Studie zeigt, dass innovative digitale Lösungen das Potenzial besitzen, die Handrehabilitation nachhaltig zu transformieren – vorausgesetzt, sie werden evidenzbasiert weiterentwickelt und systematisch in den klinischen Alltag integriert.

## Fazit für die Praxis

- Digitale Therapie-Apps reduzieren signifikant die Dauer der Arbeitsunfähigkeit nach operativ behandelten Handverletzungen.
- Patienten mit Mittelhandfrakturen profitieren besonders stark, bei deutlich schnellerer beruflicher Wiedereingliederung.
- App-gestützte Rehabilitation ergänzt die Standardtherapie durch individualisierte Übungspläne, Echtzeit-Feedback und motivierende Gamification-Elemente.
- Digitale Rehabilitationsansätze besitzen neben klinischen Vorteilen auch ein erhebliches gesundheitsökonomisches Potenzial.

## Abkürzungen

BAuA: Bundesanstalt für Arbeitsschutz und Arbeit
DAHTH: Deutsche Arbeitsgemeinschaft für Handtherapie
IG: Interventionsgruppe
KG: Kontrollgruppe
KI: Künstliche Intelligenz
MDR: Medical Device Regulation
ROM: „Range of motion“
SoC: Standardversorgung
VAS: Visuelle Analog Skala
